# Aspergillose naso-sinusienne pseudo-tumorale

**DOI:** 10.11604/pamj.2015.22.221.8116

**Published:** 2015-11-10

**Authors:** Rim Lahiani, Madiha Mahfoudhi

**Affiliations:** 1 Service ORL, Hôpital Charles Nicolle, Tunis, Tunisie; 2 Service de Médecine Interne A, Hôpital Charles Nicolle, Tunis, Tunisie

**Keywords:** Obstruction nasale, sinus maxillaire, aspergillose, nasal obstruction, maxillary sinus, aspergillosis

## Image en medicine

L'aspergillose peut se présenter sous une forme pseudo-tumorale avec destruction des parois osseuses sinusiennes, prêtant à confusion avec des lésions tumorales. L'imagerie en particulier la TDM du massif facial joue un rôle indiscutable dans le diagnostic et le bilan d'extension des différentes formes des sinusites aspergillaires. Patiente âgé de 42 ans, aux antécédents de soins dentaires récents, a consulté pour des céphalées, une cacosmie et une obstruction nasale évoluant depuis trois semaines sans notion d'altération de l’état général ni de fièvre. La rhinoscopie antérieure et l'endoscopie nasale ont révélé du pus provenant du méat moyen droit, une muqueuse nasale inflammatoire à droite, une déviation de la cloison nasale, et un cavum libre. Le reste de l'examen physique en particulier stomatologique et des aires ganglionnaires était sans anomalies. La TDM du massif facial a révélé un comblement du sinus maxillaire droit avec un aspect lysé de sa paroi médiale et une image de calcification intra-maxillaire. Cet aspect était évocateur d'un cancer, une infection bactérienne ou un lymphome. La patiente a bénéficié d'un traitement chirurgical consistant en un nettoyage des lésions et des tissus nécrosés par voie endo-nasale. Un traitement médical associant une céphalosporine de troisième génération et un aminoside lui a été instauré ainsi que des soins locaux. La présence de filaments mycéliens d'Aspergillus fumigatus à l'examen anatomo-pathologique a confirmé le diagnostic d'aspergillose pseudo-tumorale naso-sinusienne. Elle été alors traitée par amphotéricine B. L’évolution était marquée par une amélioration sur le plan clinique et endoscopique.

**Figure 1:**
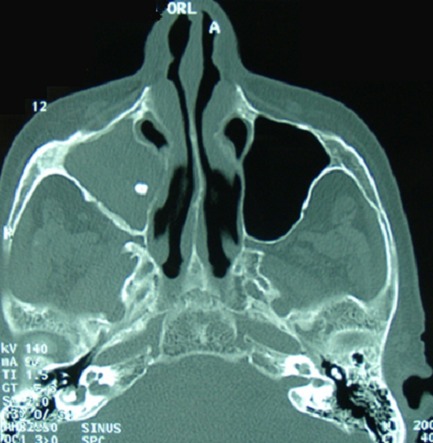
TDM du massif facial (coupe axiale): comblement du sinus maxillaire droit avec aspect lysé de sa paroi médiale et image de calcification intra-maxillaire

